# The influence of individual and partner self-esteem on relationship satisfaction: evidence from freshman using actor-partner interdependence model

**DOI:** 10.3389/fpsyg.2025.1673074

**Published:** 2025-12-29

**Authors:** Yating Yang, Yangting Wu, Jingjing Song

**Affiliations:** 1 Institute of Applied Psychology, China University of Geosciences (Wuhan), Wuhan, China; 2 School of Medicine, Hangzhou City University, Hangzhou, China

**Keywords:** actor-partner interdependence model, emotional expression patterns, individual self-esteem, partner self-esteem, relationship satisfaction

## Abstract

**Introduction:**

This study, grounded on the actor-partner interdependence model, explore how individual and partner self-esteem affect their relationship satisfaction.

**Methods:**

Three independent studies were conducted on freshmen recruited from college students’ mental health courses. Study 1 (*n* = 423) adopted a conflict situation recall task to measure participants’ own and perceived partners’ self-esteem and emotional expression. Study 2 (*n* = 145) employed the same recall task to assess these variables plus relationship satisfaction for both participants and their perceived partners. Study 3 surveyed 94 couples, measuring self-esteem, emotional expression, and relationship satisfaction for both individuals and their partners.

**Results:**

The results of Study 1 showed that individual self-esteem and perceived partner self-esteem were positively associated with individual emotional expression and perceived partner emotional expression. Study 2 further revealed that individual emotional expression was positively associated with perceived partner relationship satisfaction, and perceived partner emotional expression was positively associated with individual relationship satisfaction. The results of Study 3 indicated that female self-esteem was only positively related with her emotional expression, while male self-esteem was only positively related with his emotional expression. Moreover, female emotional expression was positively associated with male partners’ relationship satisfaction, and male emotional expression was positively associated with female partners’ relationship satisfaction.

**Discussion:**

These results suggest that individuals and their partners mutually influence each other, affecting emotional expression patterns and ultimately impacting relationship satisfaction. The findings highlight the importance of considering the mutual influence between partners in relationship interventions.

## 1 Introduction

In 2024, 6.1 million couples registered for marriage and 2.62 million couples registered for divorce in China. The persistently low marriage rate and the high divorce rate reflect the social reality of people’s marriage fear and low marital satisfaction. Marital harmony is crucial for personal happiness, family stability, and is also a prerequisite for families to actively have children. Therefore, exploring the core factors that influence relationship satisfaction has become a key issue that urgently requires attention. Self-esteem, as an individual’s overall evaluation of themselves, can affect the interaction patterns with their partners in intimate relationships. This research aims to explore the influence of individual and partner self-esteem on the relationship satisfaction through the mediating role of emotional expression. The research results have important theoretical and applied values for alleviating the marriage fear among unmarried young people, improving relationship satisfaction, promoting family harmony, and increasing the fertility rate.

### 1.1 The influence of individual self-esteem on intimate relationships

Self-esteem is an individual’s overall evaluation of themselves, related to people’s sense of value, self-acceptance, and self-respect. Individual self-esteem affects intimate relationships in three aspects: cognition (perception of intimate relationships), emotion (relationship satisfaction), and conation (including emotional and behavioral expressions). (1) Self-esteem affects the perception of intimate relationships. Individuals with low self-esteem tend to have more negative perceptions of intimate relationships. They are more prone to experiencing insecurity in relationships, are more likely to distrust and suspect their partners (Arikewuyo et al., 2021), and underestimate their partners’ love for them (Luerssen et al., 2017). These negative perceptions of intimate relationships originate from the negative information perception tendency of low self-esteem individuals. They are more sensitive to rejection information, more likely to perceive and imagine threatening information in intimate relationships, and less likely to perceive their partners’ positive responses (Marigold et al., 2020). (2) Self-esteem affects emotional and behavioral expressions in intimate relationships. There are conflicting theoretical inferences about the relationship between self-esteem and emotional and behavioral expression patterns. The risk regulation model posits that low self-esteem individuals are more likely to prioritize self-protection goals over relationship promotion goals, and may exhibit a series of behaviors that undermine intimate relationships to mitigate the pain caused by the end of the relationship (Campbell et al., 2018). For example, they may distance themselves from their partners, or even belittle their partners (Alonso-Ferres et al., 2021), be reluctant to directly express their emotions (Alonso-Ferres et al., 2021), and be unwilling to engage in self-sacrificing behaviors in intimate relationship (Luerssen et al., 2017). The commitment insurance model indicates that low self-esteem individuals are in a disadvantaged position in intimate relationships, which may trigger an exchange script. They will exhibit more emotional expressions and self-sacrificing behaviors to make their partners commit to the relationship, and ensure its stability (Righetti and Visserman, 2018). (3) Self-esteem affects relationship satisfaction. Low self-esteem individuals report lower relationship satisfaction compared to high self-esteem individuals (Chui and Leung, 2016).

### 1.2 The influence of individual and partner self-esteem on intimate relationship satisfaction

Individual self-esteem and partner self-esteem jointly affect intimate relationship satisfaction. Previous studies predominantly used the questionnaire survey method to measure the self-esteem levels of individuals and their partners concurrently. Researchers then explored the correlation relationship between the product term of the two variables, their similarity, or their difference with relationship satisfaction to indirectly reflect the interactive influence between individual self-esteem and partner self-esteem. However, relevant studies have failed to identify significant correlations (Erol and Orth, 2014; Erol and Orth, 2016; Ghaziri et al., 2019). Therefore, these data analysis method are unable to accurately reflect the interactive influence of individual self-esteem and partner self-esteem on intimate relationship satisfaction.

The actor-partner interdependence model (APIM) is a statistical model uitilized for analyzing the mutual influence between individuals and their partners in intimate relationships (Liu and Wu, 2017). APIM differentiates the impacts of individual factors (actor effects) and partner factors (partner effects) on an individual’s outcomes. In the research on intimate relationships, it can simultaneously examine the influence of individual’s traits and behaviors on their own intimate relationship satisfaction, as well as the influence of their partner’s traits and behaviors on the individual’s intimate relationship satisfaction. This model usually employs methods such as multilevel linear models or structural equation models for analysis, which can effectively isolate the unique effects of individual and partner factors, along with the possible interaction effects between them.

### 1.3 The mediating role of emotional expression in the relationship between self-esteem and relationship satisfaction

The emotional expressions may play a mediation role in the relationship between self-esteem and relationship satisfaction. Some studies have indirectly shown the influence of individual and partner self-esteem on emotional expression patterns in intimate relationships. (1) Negative emotional expression and behavioral patterns from high self-esteem individuals elicit more positive responses compared to those from low self-esteem individuals (Cortes and Wood, 2018; Peterson et al., 2019). (2) Partners with high self-esteem are more likely to perceive the positive emotional expression and behavioral patterns, and evoke positive responses. Only people who perceive their partners’ positive emotional expression and recognize their partners’ sacrifices would respond positively (LaBuda and Gere, 2021; Visserman et al., 2019).

When individuals and their partners establish positive emotional expression patterns, it can introduce a positive cycle to the intimate relationship and increase the relationship satisfaction. Previous studies have mainly indicated that individual’s positive emotional expression can improve the partner’s relationship satisfaction: (1) It meets the partner’s personal needs. Individual’s positive expression and self-sacrificing behavior directly satisfy the partner’s personal needs (Bai et al., 2022). (2) It meets the partner’s needs for intimacy and respect. A positive emotional expression pattern implies that the partner cares about and values the intimate relationship (Day and Impett, 2016). (3) It alleviates conflicts in the intimate relationship. A positive emotional expression pattern can resolve conflicts and ease tension (Gere and Impett, 2017). (4) It improves relationship satisfaction. A positive emotional expression pattern can bring positive emotional experiences to the partner, enhance partner’s personal well-being (Joel et al., 2013), relationship security (Lemay and Muir, 2016), relationship satisfaction (Marigold et al., 2007), and relationship commitment (Visserman et al., 2019).

### 1.4 Current study

Previous studies have several limitations that call for further exploration in future research: (1) Most prior research has predominantly concentrated on the impact of individual self-esteem on the cognition, emotion, and conation within intimate relationships from an individual—centered perspective. This approach has overlooked the interactive effects between individuals and their partners. Therefore, it is essential to analyze how both individual and partner self-esteem jointly influence relationship satisfaction. (2) Although a small number of studies have taken into account both individual and partner self-esteem, the data-analysis methods employed (such as interaction terms or similarity measures) are unable to directly mirror the interactional patterns between individuals and their partners in intimate relationships. Thus, it is necessary to utilize the actor-partner interdependence model to analyze the mutual influences between individuals and their partners in such relationships. (3) The interaction pattern in an intimate relationship is dynamic, immediate, and reciprocal. There is a need to explore the short-term dynamic process through which individual and partner self-esteem impact the interaction pattern in intimate relationships and relationship satisfaction.

In summary, based on the actor-partner interaction model, this study used the conflict recall task in intimate relationships and the questionnaire survey method to analyze the short-term influence process and long-term influence outcomes of individual and partner self-esteem on the emotional expression pattern in intimate relationships and relationship satisfaction.

We carried out a total of three studies. Study 1 employed the conflict-recall task in intimate relationships to analyze the short-term dynamic impact of individual self-esteem and perceived partner self-esteem on the emotional expressions of both the individual and the perceived partner. We hypothesized that individual self-esteem would be significantly and positively correlated with individual emotional expression and perceived partner emotional expression. Additionally, perceived partner self-esteem would be significantly and positively correlated with individual emotional expression and perceived partner emotional expression.

Study 2 utilized the conflict-recall task in intimate relationships to analyze the short-term dynamic influence of individual self-esteem and perceived partner self-esteem on relationship satisfaction, mediated by the emotional expressions of the individual and the perceived partner. We hypothesized that individual emotional expression would be positively associated with perceived partner relationship satisfaction, and that perceived partner emotional expression would be positively associated with individual relationship satisfaction.

Study 3 adopted the questionnaire survey method to analyze the long-term influence of individual and partner self-esteem on the interaction pattern in intimate relationships and relationship satisfaction. We hypothesized that female partners’ self-esteem would be significantly positively correlated with both their own emotional expression and male partners’ emotional expression; by the same token, male partners’ self-esteem would be significantly positively correlated with both their own emotional expression and female partners’ emotional expression. Furthermore, female partners’ emotional expression would be positively associated with male partners’ relationship satisfaction, and male partners’ emotional expression would be positively associated with female partners’ relationship satisfaction.

## 2 Study 1: the influence of individual self-esteem and perceived partner self-esteem on the emotional expression of individual and partner

### 2.1 Research methods

#### 2.1.1 Participants

We randomly selected 10 mental health course classes at a university in central China. College students in these classes who had ever been in romantic relationships were invited to participate in our investigation on a voluntary basis. After excluding 27 invalid questionnaires that were carelessly filled out, a total of 423 valid questionnaires were collected. Among the participants, 265 were male (62.6%) and 158 were female (37.4%). Additionally, 295 students (69.7%) were from urban areas, while 128 students (30.3%) were from rural areas. The average age of all participants was 18.38 years old.

#### 2.1.2 Research procedures

Permission for this study was granted by the ethics committee of the first author’s institution. Participants were recruited via the university’s mental health courses. After introducing the research content, participants first completed an informed consent form. Subsequently, they were asked to recall a conflict event that had occurred with their partners and were required to give a detailed account, covering the cause of the quarrel, the communication process between the two parties, and the way the conflict was resolved. Following this, electronic questionnaires were distributed through the Wenjuanxing online platform. Students voluntarily scanned the QR code to fill out the questionnaires, which were designed to measure their individual self-esteem, perceived partner self-esteem, individual emotional expression, and perceived partner emotional expression during the conflict event. They were rewarded with five yuan upon the completion of the entire investigation.

#### 2.1.3 Research materials

Individual self-esteem: The Rosenberg self-esteem scale (Rosenberg, 1965) was used to measure individual self-esteem. It consists of 10 items and uses a four-point scale, 1 = inconsistent with my situation, 4 = consistent with my situation. The higher the total score, the higher the individual’s self-esteem level. In this study, the Cronbach’s alpha coefficient of this scale was 0.90.

Perceived partner self-esteem: We revised some expressions of the Rosenberg self-esteem scale to measure the perceived partner self-esteem, for example, I think my partner regards himself/herself as a valuable person. The scale uses a four-point scale, 1 = inconsistent with my situation, 4 = consistent with my situation. The sum score of all the items was the final score. The higher the score, the higher the perceive partner self-esteem. In this study, the Cronbach’s alpha coefficient of this scale was 0.84.

Individual emotional expression: A self-developed individual emotional expression questionnaire was used, which consists of 3 items. For example: I am willing to tell my partner about the things I have experienced. It uses a five-point scale, 1 = extremely disagree, 5 = extremely agree. The higher the score, the higher the emotional expression in the romantic relationship. In this study, the Cronbach’s alpha coefficient of this scale was 0.90. The results of exploratory factor analysis (EFA) indicated that the Kaiser-Meyer-Olkin (KMO) measure of sampling adequacy was 0.78, and Bartlett’s test of sphericity was statistically significant (*p* < 0.001). These results demonstrated that the data of this study were suitable for factor analysis. The first extracted common factor explained 83.04% of the total variance, and the factor loadings of all items were greater than 0.6. However, due to the limited number of items, the model presented a degree of freedom of 0, making confirmatory factor analysis (CFA) infeasible.

Perceived partner emotional expression: A self-developed perceived partner emotional expression questionnaire was used. It consists of 8 items. For example: my partner’s response makes me very happy. It uses a five-point scale, 1 = extremely disagree, 5 = extremely agree. The higher the score, the higher the individual’s perceived partner emotional expression. In this study, the Cronbach’s alpha coefficient of this scale was 0.94. The results of exploratory factor analysis (EFA) showed that the Kaiser-Meyer-Olkin (KMO) measure of sampling adequacy was 0.93, and Bartlett’s test of sphericity was statistically significant (*p* < 0.001), indicating that the data of this study were suitable for factor analysis. The first extracted common factor explained 71.05% of the total variance, and the factor loadings of all items were greater than 0.6. The results of confirmatory factor analysis (CFA) demonstrated that the questionnaire had good construct validity, with all fit indices meeting acceptable criteria: chi-square to degrees of freedom ratio (CMIN/DF) = 3.52, incremental fit index (IFI) = 0.98, Tucker-Lewis index (TLI) = 0.97, normed fit index (NFI) = 0.98, comparative fit index (CFI) = 0.98, and root mean square error of approximation (RMSEA) = 0.08.

#### 2.1.4 Data analysis

First, we used the SPSS 23.0 for descriptive statistical analysis and correlation analysis. Second, we employed the AMOS 28.0 to establish a structural equation model to analyze the influence of individual and partner self-esteem on the emotional expressions of individuals and their partners. We declared data and study materials are available if readers need it.

### 2.2 Research results

#### 2.2.1 Common method bias check

We adopted Harman’s single-factor test for common method bias. The results indicated that the variance explained by the first factor was 30.30%, which was lower than 40%. Thus, no significant common method bias was detected in this study.

#### 2.2.2 Descriptive statistics and correlation analysis

As presented in Table 1, individual self-esteem had significant positive correlations with perceived partner self-esteem, individual emotional expression, and perceived partner emotional expression. Perceived partner self-esteem had significant positive correlations with individual emotional expression and perceived partner emotional expression. Additionally, individual emotional expression had a significant positive correlation with perceived partner emotional expression.

**Table 1:** Descriptive and correlation results among all variables in study 1.

Variables	*M*	*SD*	1	2	3	4
Individual self-esteem	29.46	5.40	–			
Perceived partner self-esteem	30.47	3.89	0.33^***^	–		
Individual emotional expression	10.65	3.23	0.26^***^	0.21^***^	–	
Perceived partner emotional expression	30.57	6.94	0.26^***^	0.30^***^	0.66^***^	–

***p < 0.001, **p < 0.01, *p < 0.05.

#### 2.2.3 Hypothetical model testing

We utilized AMOS 28.0 to validate the relationships among individual self-esteem, perceived partner self-esteem, and the emotional expressions of both the individual and the partner. Individual and partner self-esteem, as well as individual and partner relationship satisfaction, were treated as observed variables, whereas individual and partner emotional expressions were considered latent variables. The results demonstrated that the model exhibited a good fit, with CMIN/DF = 4.47, IFI = 0.95, TLI = 0.93, NFI = 0.93, and CFI = 0.95.

As depicted in Table 2, individual self-esteem was significantly and positively correlated with both individual emotional expression (*b* = 0.22, *p* < 0.001) and perceived partner emotional expression (*b* = 0.19, *p* < 0.001). Similarly, perceived partner self-esteem was significantly and positively correlated with individual emotional expression (*b* = 0.15, *p* < 0.01) and perceived partner emotional expression (*b* = 0.24, *p* < 0.001). These results corroborate all the research hypotheses proposed in this study.

**Table 2:** The relationship between individual self-esteem, perceived partner self-esteem and emotional expression of individual and partner.

Influence path	β	*S. E.*	*C. R.*	*P*
Individual emotional expression ← Individual self-esteem	0.22	0.01	4.27	<0.001
Perceived partner emotional expression ← Individual self-esteem	0.19	0.01	3.76	<0.001
Individual emotional expression ← Perceived partner self-esteem	0.15	0.01	2.81	<0.01
Perceived partner emotional expression ← Perceived partner self-esteem	0.24	0.01	4.80	<0.001

## 3 Study 2: the influence of individual self-esteem and perceived partner self-esteem on relationship satisfaction through emotional expression

### 3.1 Research methods

#### 3.1.1 Participants

We randomly selected two mental health course classes at a university located in central China. College students who had ever been in romantic relationships were invited to participate in this investigation on a voluntary basis. After excluding 5 invalid questionnaires that were carelessly filled out, a total of 145 valid questionnaires were collected. Among the participants, 92 were male (63.4%) and 53 were female (36.6%). Additionally, 102 students (70.4%) hailed from urban areas, while 43 students (29.6%) were from rural areas. The average age of all participants was 18.79 years old.

#### 3.1.2 Research procedures

Permission for this study was granted by the ethics committee of the first-author’s institution. Participants were recruited via the university’s mental health courses. After the research content was introduced, participants first completed an informed-consent form. Subsequently, participants were asked to carefully recall a typical quarrel event in their intimate relationships and record it in writing. The recorded content encompassed the cause of the quarrel, their communication manner at that time, and participants were also required to subjectively evaluate their own expression patterns and their partners’ response patterns. Once the participants had completed the conflict-scenario task, electronic questionnaires were distributed through the Wenjuanxing online platform. Students voluntarily scanned the QR code to fill out the questionnaires, which were designed to measure their self-esteem, emotional expression, and relationship satisfaction, along with perceived partner self-esteem, perceived partner relationship satisfaction, and perceived partner emotional expression. They were rewarded with five yuan upon the completion of the entire investigation.

#### 3.1.3 Research materials

Individual self-esteem: same as Study 1. The Cronbach’s alpha coefficient was 0.87 in current study.

Perceived partner self-esteem: same as Study 1. The Cronbach’s alpha coefficient was 0.78 in current study.

Individual emotional expression: The intimate relationship supportive behavior scale developed by Overall et al. (2010) was selected. This questionnaire consists of 22 items and three dimensions: nurturant support, action-facilitating support, and negative support. The nurturant support subscale includes emotional support (e.g., I express love and affection to my partner) and esteem support (e.g., Compliments or says positive things about the partner and/or emphasizes the partner’s abilities to bring about change), and it contains 8 items. In the current study, we used the nurturant support subscale to measure individual emotional expression. Participants were required to evaluate the frequency of their emotional expression to their partners in the conflict event. A 5-point Likert scale was used, 1 = never, 5 = very often. A higher total score indicates a higher level of individual emotional expression. The Cronbach’s alpha coefficient of this scale in this study was 0.92.

Perceived partner emotional expression: The above individual emotional expression questionnaire was modified to measure perceived partner emotional expression. For example, I think my partner express love and affection to me. A 5-point Likert scale was used. A higher total score indicates a higher level of perceived partner emotional expression. The Cronbach’s alpha coefficient was 0.89 in current study.

Individual relationship satisfaction: The relationship quality index (Patrick et al., 2007; Norton, 1983) was adopted to measure individual relationship satisfaction. It consists of 6 items. For example, my relationship with my partner makes me feel happy. A 7-point Likert scale was used, 1 = completely disagree, 7 = completely agree. A higher score indicates a higher level of relationship satisfaction. The Cronbach’s alpha coefficient of the questionnaire in this study was 0.91.

Perceived partner relationship satisfaction: The individual relationship satisfaction questionnaire was revised to measure perceived partner relationship satisfaction. It consists of 6 items. For example: My partner thinks this relationship makes him/her feel happy. A higher score indicates a higher level of perceived partner relationship satisfaction. The Cronbach’s alpha coefficient of the questionnaire in this study was 0.93.

#### 3.1.4 Data analysis

First, the SPSS 23.0 was used for descriptive statistical analysis and correlation analysis. Second, the AMOS 28.0 was used to establish a structural equation model to examine the relationship between individual and partner self-esteem and relationship satisfaction of individual and partner emotional expression. We declared data and study materials are available if readers need it.

### 3.2 Research results

#### 3.2.1 Common method bias check

Harman’s single-factor test was employed to examine for common method bias. The results indicated that the variance explained by the first factor was 25.89%, which was below 40%. Thus, no significant common method bias was detected in this study.

#### 3.2.2 Descriptive statistics and correlation analysis

As presented in Table 3, individual self-esteem was significantly and positively correlated with perceived partner self-esteem, individual relationship satisfaction, perceived partner relationship satisfaction, individual emotional expression, and perceived partner emotional expression. Perceived partner self-esteem was significantly and positively correlated with individual relationship satisfaction, perceived partner relationship satisfaction, individual emotional expression, and perceived partner emotional expression.

**Table 3:** Descriptive and correlation results among all variables in study 2.

Variables	*M*	*SD*	1	2	3	4	5	6
Individual self-esteem	29.66	4.40	–					
Perceived partner self-esteem	29.85	3.28	0.50^***^	–				
Individual relationship satisfaction	23.28	4.87	0.21^**^	0.38^***^	–			
Perceived partner relationship satisfaction	23.41	4.69	0.25^**^	0.37^***^	0.87^***^	–		
Individual emotional expression	26.37	4.31	0.39^***^	0.35^***^	0.43^***^	0.39^***^	–	
Perceived partner emotional expression	26.58	4.89	0.35^***^	0.26^**^	0.58^***^	0.57^***^	0.67^***^	–

***p < 0.001, **p < 0.01, *p < 0.05.

#### 3.2.3 Hypothetical model testing

AMOS 28.0 was utilized to validate the relationships among individual self-esteem, perceived partner self-esteem, and the relationship satisfaction of both the individual and the partner. Self-esteem and relationship satisfaction were treated as observed variables, whereas emotional expressions were regarded as latent variables. The results demonstrated that the model had a good fit, with CMIN/DF = 1.944, IFI = 0.92, TLI = 0.91, CFI = 0.92, and RMSEA = 0.08.

As depicted in Table 4, individual self-esteem was significantly and positively correlated with individual emotional expression (*b* = 0.30, *p* < 0.01) and perceived partner emotional expression (*b* = 0.22, *p* < 0.01). Similarly, perceived partner self-esteem was significantly and positively correlated with individual emotional expression (*b* = 0.25, *p* < 0.05) and perceived partner emotional expression (*b* = 0.16, *p* < 0.05). Additionally, individual emotional expression was positively associated with perceived partner relationship satisfaction (*b* = 0.40, *p* < 0.001), and perceived partner emotional expression was positively associated with individual relationship satisfaction (*b* = 0.59, *p* < 0.001). These results corroborate all the research hypotheses proposed in this study.

**Table 4:** The relationship between individual self-esteem, perceived partner self-esteem and the relationship satisfaction of individual and partner.

Influence path	β	*S. E.*	*C. R.*	*P*
Individual emotional expression ← Individual self-esteem	0.30	0.01	2.99	<0.01
Perceived partner emotional expression ← Individual self-esteem	0.22	0.01	2.67	<0.01
Individual emotional expression ← Perceived partner self-esteem	0.25	0.01	2.55	<0.05
Perceived partner emotional expression ← Perceived partner self-esteem	0.16	0.02	1.96	<0.05
Perceived partner relationship satisfaction ← Individual emotional expression	0.40	0.96	4.22	<0.001
Individual relationship satisfaction ← Perceived partner emotional expression	0.59	0.55	7.72	<0.001

## 4 Study 3 the influence of individual and partner self-esteem on relationship satisfaction: based on actor-partner interdependence model

### 4.1 Research methods

#### 4.1.1 Participants

We are distributing recruitment posters at a university in central China. A total of 94 heterosexual couples volunteered to take part in this investigation. The female participants had an average age of 22.31 years (*SD* = 2.86, with ages ranging from 17 to 31 years). Regarding educational attainment, 4 held doctoral degrees, 62 were master’s degree holders, and 28 were undergraduate students. The male participants had an average age of 22.79 years (*SD* = 3.23, with ages ranging from 17 to 34 years). Among them, 10 had doctoral degrees, 65 were master’s degree holders, and 19 were undergraduate students. The average duration of their romantic relationships was 2.47 years.

#### 4.1.2 Research procedures

Ethical approval for this study was secured from the ethics committee of the first author’s institution. Recruitment of couples was carried out by posting posters at the university. Questionnaires were disseminated via the Wenjuanxing online platform. Students voluntarily scanned the QR code to complete the questionnaires. Prior to commencing, after the research content was explained, participants were asked to fill out an informed consent form. Additionally, they were required to provide their partners’ mobile phone numbers. This information served as the basis for ensuring accurate pairing of couples. Participants were then tasked with answering questions related to individual self-esteem, individual relationship satisfaction, individual emotional expression, perceived partner self-esteem, perceived partner relationship satisfaction, perceived partner emotional expression, and demographic details. As an incentive, they received a monetary reward of 10 yuan upon successfully finishing the entire investigation.

#### 4.1.3 Research materials

Individual self-esteem: Similar to Study 1, the Cronbach’s alpha coefficient of the individual self-esteem scale in this study was 0.88.

Individual emotional expression: Similar to Study 2, the Cronbach’s alpha coefficient of the individual emotional expression subscale for participants was 0.88.

Individual relationship satisfaction: Similar to Study 2, the Cronbach’s alpha coefficient of the questionnaire in this study was 0.84.

#### 4.1.4 Data analysis

First, the SPSS 23.0 was used for preliminary data analysis such as descriptive statistics and correlation analysis to understand the basic relationships and distribution characteristics among variables. Second, the AMOS 28.0 was used to establish an actor-partner interdependence model to explore and verify the complex relationships among variables. We declared data and study materials are available if readers need it.

### 4.2 Research results

#### 4.2.1 Common method bias check

Harman’s single-factor test was used to test the common method bias. The results showed that the variance explained by the first factor was 25.08%, which was lower than 40%. Therefore, there is no obvious common method bias in this study.

#### 4.2.2 Descriptive statistics and correlation analysis

As presented in Table 5, female self-esteem was significantly correlated with female relationship satisfaction, male self-esteem, and male relationship satisfaction. Male self-esteem was significantly and positively correlated with female self-esteem, female emotional expression, male relationship satisfaction, and male emotional expression.

**Table 5:** Descriptive and correlation results among all variables in study 3.

Variables	*M*	*SD*	1	2	3	4	5	6
Female self-esteem	32.87	5.57						
Female emotional expression	49.62	4.89	0.16					
Female relationship satisfaction	36.30	4.31	0.38^***^	0.21^*^				
Male self-esteem	33.72	4.96	0.43 ^***^	0.31^**^	0.19			
Male emotional expression	49.62	4.89	0.16	0.99^**^	0.21^*^	0.31^**^		
Male relationship satisfaction	37.20	4.52	0.22^*^	0.68^***^	0.35^***^	0.27^**^	0.68^***^	

***p < 0.001, **p < 0.01, *p < 0.05.

#### 4.2.3 Hypothetical model testing

Actor-partner interdependence model was employed to verify the relationships among self-esteem, partner self-esteem, and the relationship satisfaction of both the female and the male partner. Self-esteem and relationship satisfaction were observed variables, while emotional expression were latent variables. As shown in Table 6, the results indicated that female self-esteem was not significantly related to the emotional expression of the male partner (*b* = 0.06, *p* = 0.58), and male self-esteem was not significantly related to the emotional expression of the female partner (*b* = 0.02, *p* = 0.89), demonstrating the partner effect was not significant. Moreover, male self-esteem was positively associated with male expressional expression (*b* = 0.34, *p* < 0.01), female self-esteem was positively associated with female expressional expression (*b* = 0.31, *p* < 0.05), demonstrating a significant actor effect. In addition, male expressional expression was positively associated with female relationship satisfaction (*b* = 0.25, *p* < 0.05), female expressional expression was positively associated with male relationship satisfaction (*b* = 0.41, *p* < 0.001). The partner effect between emotional expression and relationship satisfaction was significant.

**Table 6:** The relationship between self-esteem and relationship satisfaction: based on actor-partner interaction model.

Influence path	*Β*	*S. E.*	*C. R.*	*P*
Female emotional expression ← Female self-esteem	0.31	0.02	2.58	<0.001
Male emotional expression ← Male self-esteem	0.34	0.01	2.80	<0.01
Male emotional expression ← Female self-esteem	0.06	0.01	0.55	0.58
Female emotional expression ← Male self-esteem	0.02	0.02	0.14	0.89
Male relationship satisfaction ← Female emotional expression	0.41	0.67	3.80	<0.001
Female relationship satisfaction ← Male emotion expression	0.25	0.79	2.22	<0.05

## 5 Discussion

The results of Study 1 indicated that both individual self-esteem and perceived partner self-esteem were positively correlated with individual emotional expression and perceived partner emotional expression. Study 2 further demonstrated that individual emotional expression was positively related to perceived partner relationship satisfaction, and perceived partner emotional expression was positively related to individual relationship satisfaction. The findings of Study 3 revealed that female self-esteem was only positively associated with her own emotional expression and not significantly correlated with the male partner’s emotional expression. Conversely, male self-esteem was positively associated with his own emotional expression and not significantly associated with the female partner’s emotional expression. Moreover, female emotional expression was significantly linked to male partner’s relationship satisfaction, and male emotional expression was significantly linked to female partner’s relationship satisfaction.

### 5.1 The influence of individual and partner self-esteem on relationship satisfaction

Study 1 and 2 discovered that, in specific conflict situations, individual self-esteem and perceived partner self-esteem were both positively associated with individual emotional expression and perceived partner emotional expression. Individuals with high self-esteem place greater emphasis on positive information in heterosexual relationships, hold more positive perceptions of intimate relationships and partners’ response (Marigold et al., 2020), feel more secure in intimate relationships, and trust their partners more (Arikewuyo et al., 2021; Luerssen et al., 2017). Moreover, individuals with high self-esteem are more inclined to prioritize relationship-promotion goals over self-protection goals. They are more willing to express positive emotions toward their partners in intimate relationships (Alonso-Ferres et al., 2021) and are willing to make sacrifices to maintain the intimate relationship (Luerssen et al., 2017), which corroborates the risk—regulation model.

Study 2 found that individual emotional expression was positively related to the perceived partner relationship satisfaction, and perceived partner emotional expression was positively related to individual relationship satisfaction. Previous research has pointed out that positive emotional expressions and self-sacrificing behaviors typically evoke gratitude from the partner and trigger positive responses from the partner (Joel et al., 2013), which will further enhance the perceived partner relationship satisfaction. Simultaneously, the perceived partner emotional expression provides the individual with clues regarding the emotional atmosphere in the relationship and the partner’s attitude. Based on the reception and interpretation of these clues, the individual forms an overall judgment of the relationship, thus significantly influencing their satisfaction in the relationship. Previous research has indicated that when the partner expresses understanding and gratitude for the individual’s sacrifice, it makes the sacrificer feel that their sacrifice is valuable and worthwhile, which in turn elicits positive responses (Oishi et al., 2019) and improves their relationship satisfaction.

Regarding the paired-couple data, female self-esteem was not significantly associated with male emotional expression, and male self-esteem was not significantly associated with female emotional expression. This differs from the results of Study 1 and 2, and may be attributed to the following reasons: (1) Studies 1 and 2 assessed perceived partner self-esteem, perceived partner relationship satisfaction, and perceived partner emotional expression—rather than partners’ actual self-esteem, relationship satisfaction, and emotional expression. In contrast, Study 3 collected dyadic data. The actor-partner interdependence model was properly specified for Study 3, the effects observed in Studies 1 and 2 stemmed primarily from perceptual biases driven by the actor’s own self-esteem, rather than from genuine partner effects. (2) Study 1 and 2 employed the conflict-scenario recalling task. The conflict scenario represents a typical two-person interaction situation where the two individuals mutually influence each other. Individual self-esteem affects the emotional expression of their partner, and the partner’s self-esteem affects the individual’s emotional expression. However, Study 3 used the questionnaire survey method and focused on the fixed interaction patterns formed in long-term interactions. In long-term interactions, people are more accustomed to being egocentric and concentrating on their own personal needs, and their attention to the partner’s self-esteem and emotions diminishes. Therefore, the partner effect is not significant.

### 5.2 Implication

In practice, relationship counselors and therapists can leverage these findings to design more effective intervention programs, guiding couples to understand the mutual influence mechanism through which self-esteem impacts emotional expression and relationship satisfaction. Therapists should place equal emphasis on improving individuals’ self-esteem and developing their emotional expression skills. For instance, couples can be trained to recognize signs of low self-esteem in their partners and respond in ways that encourage rather than suppress emotional sharing.

### 5.3 Limitation

The following limitations exist in current study and require further improvement in future research. (1) The participants across all three studies were Chinese freshmen. The sample was characterized by a relatively young age, cultural specificity, and gender imbalance, which further constrains the generalizability of the research findings. (2) In Study 1, the measurements of the individual emotional expression and the perceived partner emotional expression were conducted using self-developed questionnaires. Even though these questionnaires demonstrated high internal consistency and good construct validity, they lack test–retest validity, criterion-related validity, and content validity—limitations that undermine the overall validity and scientific rigor of the self-developed questionnaires. (3) There were inconsistencies in the measurement tools adopted across the three studies. Specifically, Study 1 utilized a self-developed 3-item emotional expression scale, whereas Studies 2 and 3 employed the scale developed by Overall et al. Such discrepancies in measurement instruments may compromise the comparability of results across the three studies, thereby impeding the derivation of unified conclusions regarding the effects of emotional expression. (4) In Study 2, only one partner was surveyed, and the perceived partner self-esteem, perceived partner relationship satisfaction, and perceived partner emotional expression were measured. Thus, these results are essentially actor-effects on perception, not true partner effects. In future research, couples should be invited to recreate the scenes of their past quarrels, and then measured both partners. (5) The validation evidence for the task-based procedures used to elicit emotional or cognitive responses prior to questionnaire administration was not sufficiently provided, raising questions about whether these tasks effectively induced the intended psychological states.

## Data Availability

The raw data supporting the conclusions of this article will be made available by the authors, without undue reservation.
